# Molecular Evaluation of the Effects of *FLC* Homologs and Coordinating Regulators on the Flowering Responses to Vernalization in Cabbage (*Brassica oleracea* var. *capitata*) Genotypes

**DOI:** 10.3390/genes15020154

**Published:** 2024-01-24

**Authors:** Ju-Young Ahn, Saminathan Subburaj, Fanzhuang Yan, Jian Yao, Ajithan Chandrasekaran, Kyoung-Gu Ahn, Geung-Joo Lee

**Affiliations:** 1 Department of Horticulture, Chungnam National University, Daejeon 34134, Republic of Korea; wnduds357@naver.com (J.-Y.A.); sami_plantbio86@yahoo.co.in (S.S.); ajithancbiotech@gmail.com (A.C.); 2 Department of Smart Agriculture Systems, Chungnam National University, Daejeon 34134, Republic of Korea; yanfanzhuang@126.com (F.Y.); yaojiansdau@163.com (J.Y.); 3 Joen Seed Co., Ltd., Goesan 28051, Republic of Korea; kyounggu.ahn@joeunseeds.com

**Keywords:** *BoFLC*, cabbage flowering, flowering regulator, structural variation, vernalization

## Abstract

The flowering loci of cabbage must be understood to boost their productivity. In this study, to clarify the flowering mechanisms of cabbage, we examined the three flowering repressors *BoFLC1*, *2* and *3*, and the flowering regulators *BoGI*, *BoCOOLAIR*, and *BoVIN3* of early (CAB1), middle (CAB3), and late (CAB5) flowering cabbage genotypes. Analysis of allele-specifically amplified genomic DNA and various sequence alignments demonstrated that maximal insertions and deletions influenced cabbage flowering behavior, notably in CAB3 and CAB5. Phylogenetic studies showed that *BoFLC1*, *2*, and *3* in the CAB1, 3, and 5 genotypes had the highest homologies to other *Brassica* species, with CAB3 and 5 the most similar. Although CAB3 and CAB5 have comparable genetic patterns, flowering repressors and flowering regulators were investigated individually with and without vernalization to determine their minor flowering differences. The expression investigation revealed that vernalized CAB5 downregulated all *BoFLC* genes compared to CAB3 and, in contrast, CAB3 exhibited upregulated *BoCOOLAIR*. We hypothesized that the CAB3 *BoFLC* locus’ additional insertions may have led to *BoCOOLAIR* overexpression and *BoFLC* downregulation. This study sheds light on cabbage genotypes—particularly those of CAB1 and CAB5—and suggests that structural variations in *BoFLC2* and *3* bind flowering regulators, such as *COOLAIR*, which may affect cabbage flowering time.

## 1. Introduction

In plants, regulation of flowering time is one of the most important events that significantly affects the synchronization of reproduction under favorable environmental conditions. Floral transition, which encompasses the vegetative-to-reproductive phase, is a vital developmental process that governs the timing of reproduction. The role of environmental (photoperiod and temperature) and endogenous factors (autonomous, gibberellins [GA], and aging) in flowering have been characterized in *Arabidopsis* [1,2,3,4]. Floral transitions like vernalization induced by cold temperature are widely present in long-day biennial and winter plants. This vernalization pathway comprises many genes that act as transcriptional and epigenetic silencing mechanisms to form a complex regulatory network that controls flowering time.

Brassicaceae are some of the most economically important crop species in horticulture and are cultivated mainly for their edible seeds, stems, leaves, and flowers. The economic value of Brassicaceae crops is heavily influenced by vernalization, which occurs when the plants are forced to bloom after experiencing cold temperatures. The early spring harvest of cabbage (heads) occurs in cultivars that bolt late and have high vernalization needs. For example, cabbage (*Brassica oleracea* var. *capitata*), a plant of this vernalization-induced flowering type, is insensitive to cold signals until it reaches a certain size (critical mass) and needs prolonged exposure to cold temperatures to flower. This might cause a delay in the breeding cycle [5]. Therefore, breeding late-flowering cabbages that are resistant to cold temperatures and bolting is of considerable interest to breeders and researchers [6,7]. Important vernalization-related molecular components such as flowering-related genes such as *FLOWERING LOCUS C (FLC)*, *FRIGIDA (FRI)*, and *VERNALIZATION INSENSITIVE 3 (VIN3)* are evolutionarily conserved between the spring and winter forms of *Arabidopsis* and *Brassica* crops [8,9,10,11,12]. The natural variation in flowering time between spring and winter forms is mostly associated with allelic variations in paralogs of the *FLC* locus, which has already been characterized in many *Brassica* species, including *B*. *oleracea* [13,14,15] *B*. *rapa* [16,17], and *B*. *napus* [18,19].

The *BoFLC1*, *BoFLC2*, and *BoFLC3* genes, as well as the *BoFLC5* pseudogene, are present in the reference genome of *B. oleracea* at Chromosome 9 (C9), C2, C3, and C3, respectively. [11,20,21]. In earlier studies, *BoFLC2* was found to be the only main functional gene responsible for floral transitions [11,13]. Supporting this, mutations in *BoFLC2* cause a loss of function in annual cauliflower varieties [14]. A recent genomic study showed that three *BoFLC*s delayed flowering in *B. oleracea* and suggested that these paralogs were functionally conserved as floral repressors [22]. However, the investigated mRNA expression characteristics revealed that *BoFLC1* declined less markedly than the highly repressed genes *BoFLC2* or *3* during vernalization [22]. In addition, an insertion mutation in intron 2 of *BoFLC1* and its rapid downregulation under vernalization was strongly associated with the vernalization-dependent phenotype of an early flowering cabbage line. Allelic diversity and variations in *FRI* have also been closely linked to flowering or heading date variations in *B*. *oleracea* [23,24] and *B*. *napus* [25].

The formation of flower meristems and organ determination during vernalization-dependent floral signal integration is influenced by the chain regulation of *FRI*, *FLC*, *FLOWERING LOCUS T (FT)*, and MADS-box transcription factor proteins such as SUPPRESSOR of OVEREXPRESSION of CONSTANS1 (SOC1) and LEAFY (LFY) [26,27]. In the autonomous and vernalization pathways, the antisense regulator *Cold induced long antisense intragenic RNA* (*COOLAIR)* along with *GIGANTEA (GI)* and *VIN3* induce flowering by altering the expressions of *FLC*, *FT*, and *SOC1* [28]. GI, a circadian clock-associated protein, is involved in various physiological processes, including the regulation of flowering time [29]. *GI* gene positively regulates *CONSTANS (CO)* and *FT*, suggesting a role in floral transition [30,31].

Under non-vernalization conditions, *FLC* expression is increased by an activator called *FRI*, which recruits chromatin modification factors to the *FLC* chromatin, thereby postponing flowering time. Natural antisense transcripts (NATs) play pivotal roles in the epigenetic silencing of *FLC* [32]. Vernalization induces the transcription of noncoding sense and antisense transcripts of *Cold-assisted intronic non-coding RNA (COLDAIR)* and *COOLAIR* from the first intronic and multi-exonic or promoter-adjacent 3′ regions in the *FLC* locus of *Arabidopsis*, respectively [33,34]. *COLDAIR* aids in the addition of repressive factors to *FLC* chromatin through its interaction with *VIN3* by recruiting the PRC2-like complex, a group of polycomb proteins, to the histones of *FLC* [34]. Antisense transcripts of *COOLAIR* are produced independently of this but before *FLC* expression is decreased by the *PRC2*-like complex, repressing the sense transcripts of *FLC* [32].

To date, variations in the flowering times (early, mid, and late) and vernalization sensitivities among cabbage cultivars have been inadequately investigated. In this study, we characterized three *BoFLC* functional members—*BoFLC1*, *BoFLC2*, and *BoFLC3*—in early (CAB1)-, mid (CAB3)-, and late-flowering (CAB5) cabbage cultivars. We found that allelic variations in the *BoFLC* locus could alter vernalization sensitivity between cabbage cultivars. This also suggests that these allelic nucleotide variations may influence the epigenetic silencing of *FLC*, as noted in *Arabidopsis.* Therefore, we propose that the identification of cis polymorphisms in *BoFLC1*, *BoFLC2*, and *BoFLC3* provides beneficial information for breeding late-flowering and cold-resistant *Brassica* crops.

## 2. Materials and Methods

### 2.1. Plant Materials and Growth Conditions

Three inbred lines or commercial cultivars i.e., 20FL-CAB1 (early-flowering, 140–150 days), 20FL-CAB3 (mid-flowering, 160–170 days), and 20FL-CAB5 (late-flowering, ≥190 days), with distinct flowering times (varying from 40–45 days) were derived from a domestic seed company (Joeun Seeds Co., Ltd., Goesan, Chungbuk, Republic of Korea) (Table S1). Seeds of all lines were sown and cultivated in a greenhouse (25 and 20 ˚C under 16 and 8 h light and dark conditions, respectively) for 30 days in April 2020 and then transplanted to the field. Sixty days after field transplantation, the head portions of the cabbages were removed and transferred to plastic pots (30 cm × 25 cm) containing a mixture of cocopeat and soil in a 1:1 ratio. A set of pots without vernalization (control) was allowed to grow under greenhouse conditions with 16 h of light at 25 °C and 8 h of dark at 18 °C. For vernalization treatment, pots were transferred to TOGA UGSR01 incubators (TOGA clean system, Yuseong, Daejeon, Republic of Korea) maintained at 4 °C with a 10 h/14 h light/dark cycle for 11 weeks. Leaf samples were collected from young leaves of three replicates of the three respective types of cabbage grown under both the control and vernalization conditions each week (0–11 weeks of vernalization). The collected leaf samples were quickly frozen in liquid nitrogen and stored at −80 °C for further use.

### 2.2. Genomic DNA Extraction, PCR Amplification, and Sequencing of BoFLC Homologs

Genomic DNA was extracted from young leaves using a WizPrep Plant DNA Mini Kit (WizBiosolutions, Seongnam, Gyeonggi, Republic of Korea). According to the gene sequences of *BoFLC1* (*AM231517.1*), *BoFLC2* (*AY306124.1*), and *BoFLC3* (*AY306125.1*) deposited in GenBank, allele-specific (AS) PCR primers (Table S2) were designed and used to amplify *BoFLC-*encoding genes in cabbage. PCR amplifications were conducted with gene-specific primers in a 50 μL reaction volume containing 100 ng of DNA, 0.5 μM of both forward and reverse oligos, 0.5 μM dNTP, 10x Ex Taq buffer, 0.8 units of Ex Taq polymerase (Takara, Shimogyo, Kyoto, Japan) and autoclaved water. PCR reactions were performed on a thermal cycler using the following program: one cycle of 30 s at 98 °C; 30 cycles of 30 s at 98 °C, 30 s at 60 °C, 3 min 30 s at 72 °C; and one cycle of 15 min at 72 °C. The PCR products were resolved on a 1% agarose gel in 1x TBE buffer, then stained with ethidium bromide and visualized under a UV transilluminator. The expected amplicons were excised from the gel and purified using a LaboPass^TM^ Gel Extraction Kit (Cosmo Genetech, Seongdong, Seoul, Republic of Korea) as recommended by the manufacturer. To sequence the purified amplicons, 2000 bp contig primers flanking the entire gene were designed for each *BoFLC* locus using the Primer3Plus online tool. PCR amplicons were sequenced using an ABI3730XL sequencer (Macrogen Co., Gangnam, Seoul, Republic of Korea).

### 2.3. Sequence Analyses and Phylogenetic Analysis of BoFLC Homologs

The *BoFLC1* genomic DNA sequences obtained in this study were deposited in the National Agricultural Biotechnology Information Center (NABIC; https://nabic.rda.go.kr/ (accessed on 20 April 2020)) database under the following numbers: NU-1420-000001 (*BoFLC1* from CAB1) and NU-1425-000001 (*BoFLC1* from CAB5). The sequencing results of nucleotide contigs were assembled, and comparative analysis among other *BoFLC* homologs to identify sequence variations (insertions and deletions) was conducted using the BioEdit (version 7.2.5) sequence alignment software (Ibis Biosciences, Carlsbad, CA, USA). The intron–exon organization of *BoFLC* homologs was predicted using the Gene Structure Display Server (GSDS2.0; http://gsds.cbi.pku.edu.cn/, version 2.0 (accessed on 12 April 2020)) by aligning the genomic sequences with their respective CDS sequences.

Multiple sequence alignments of the deduced amino acid sequences of the identified *BoFLC* homologs were performed using CLUSTAL multiple sequence alignment in MUSCLE with the default parameters (http://www.ebi.ac.uk/Tools/msa/muscle/, version 3.8 (accessed on 5 April 2020)). To analyze the evolutionary relationships among *FLC* homologs from different *Brassica* species, a maximum likelihood tree and neighbor-joining tree (1000 replicates) were constructed using a dataset of *FLC* genes containing 31 deduced amino acid sequences, including those from cabbage in this study and those from other *Brassica* species (*B*. *rapa*, *B*. *nigra*, *B*. *oleracea*, and *B*. *napus*) reported in previous studies (Table S3) [15,35]. The translated sequences of the whole coding regions of *FLC* were aligned using the MUSCLE method, and a tree was constructed using the neighbor-joining (NJ) method with MEGA version 10.0.5 and bootstrap analysis of 1000 replicates.

### 2.4. Extraction of mRNA and Expression Profiling of Flowering Genes

The leaf samples of three replicates from the two individual treatments (vernalized and non-vernalized) were subjected to total RNA isolation each week of treatment using the GeneAll Hybrid RNA purification kit (GeneAll Biotechnology, Songpa, Seoul, Republic of Korea), following the manufacturer’s instructions. A total of 1.5 μg of RNA was used to synthesize complementary DNA (cDNA) using the PrimeScript^®^ RT reagent with a gDNA eraser kit (Takara, Shimogyo, Kyoto, Japan), following the directions provided by the manufacturer. To determine the expression profile of the flowering loci (*BoFLC1*, 2, and 3), and flowering regulator genes (*BoGI*, *BoCOOLAIR*, and *BoVIN3*), gene-specific primers were generated for qRT-PCR analysis using Primer3Plus (Table S2). Gene expression was determined using the Actin1 gene internal control system with the corresponding primers (Table S2). The qRT-PCR was conducted using a reaction mixture of 13 μL, consisting of 2 μL of cDNA template, 6 μL of 2x SYBR green Q master LaboPassTM (COSMOgenetech, Seongdong, Seoul, Republic of Korea), and 0.5 μM of gene-specific forward and reverse oligonucleotide primers. The CFX96 Real-Time PCR Detection System (Bio-Rad Richmond, Hercules, CA, USA) was used with the following parameters: initial denaturation cycle at 95 °C for 3 min, followed by 40 cycles of denaturation at 95 °C for 15 s, annealing at 60 °C for 20 s, and extension at 72 °C for 15 s. A cycle at 65 °C for 5 s was included, and a final cycle at 95 °C for 2 s was performed to detect primer specificity based on melt curve analysis. Three independent replicates were conducted to determine the relative expression levels of target gene transcripts. The reference gene *β-Actin* was used as an internal normalization control and normalization was performed using the 2−ΔΔCT method [36] using CFX Manager 2.3 software (Bio-Rad Richmond, Hercules, CA, USA). The 5th week untreated and cold-treated CAB1 cultivar (early flowering) samples were considered the control (the value of 1), or reference, samples for determining the expression values of target genes of non-treatment control and treated CAB3 (mid flowering) and CAB5 (late flowering) plants. The obtained data were further subjected to statistical analysis.

### 2.5. Statistical Analysis

To analyze statistically significant differences in the relative expression of genes, an ANOVA with Duncan’s multiple range test was performed using SPSS version 21.0.

## 3. Results

### 3.1. Molecular Characterization of BoFLC in Three Different Cabbage Lines with Divergent Flowering Times

**Figure 1:**
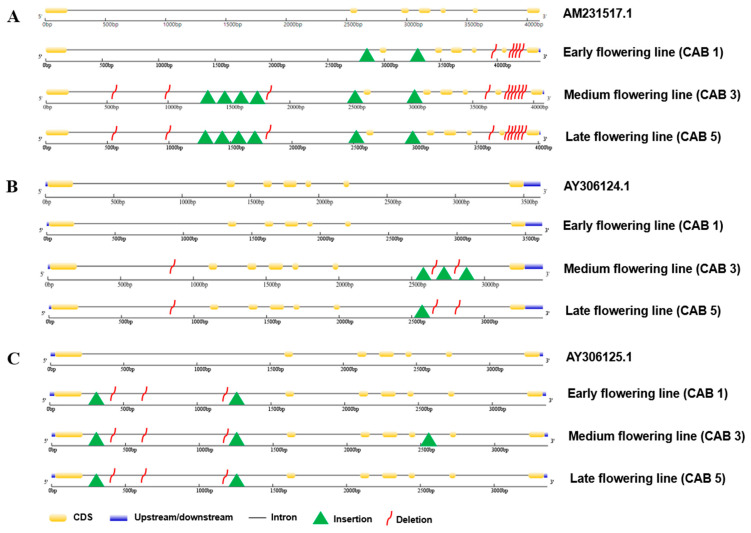
Insertion and deletion profiles of the *BoFLC* genes including *BoFLC1* (**A**), *BoFLC2* (**B**), and *BoFLC3* (**C**) in the genomic DNA of the three flowering lines CAB1, 3, and 5 along with the structure of the reference gene *AY306124.1*.

Genomic DNA from approximately 3361 to 4384 bp for *BoFLC1*, *BoFLC2*, and 160 *BoFLC3* along with coding sequences (CDSs) and an untranslated region of 594 bp were sequenced from cabbage lines with varying flowering times (Figure 1; Table S1). Various deletions and insertions were identified in the various introns of the respective cabbage DNA sequences compared with the DNA sequence of the reference, *AY306124* (NCBI Gene bank ID).

The *BoFLC1* gene sequence in all three flowering cabbage lines showed an insertion (687 and 67 bp) in intron 1 between 2500 and 3000 bp and another insertion in intron 2 between 3000 and 3500 bp. CAB3 and CAB5 exhibited four additional insertions (3, 9, 9, and 13 bp) between 1000 and 2000 bp, unlike CAB1. In *BoFLC2*, while CAB1 remained unchanged genotypically, CAB3 and CAB5 exhibited a similar type of insertion at approximately 2500 bp (9, 2, and 1 bp) in the sixth intron; furthermore, CAB3 carried two additional insertions within the sixth intron between 2500 and 3000 bp (9 bp). *BoFLC3* exhibited two insertions (13 and 21 bp) in the first intron within 1000 bp in all three flowering types; consequently, the fifth intron of CAB3 possessed an additional insertion spanning from 2000 to 3000 bp (15 bp; Figure 1A–C).

In terms of the deletions in *BoFLC* genes in the respective cabbage flowering lines, *BoFLC1* exhibited one deletion between 3500 and 4000 (70 bp) in the fifth intron and four deletions (40, 13, 29, and 78 bp) in the sixth intron of CAB1. The CAB3 and CAB5 lines exhibited three analogous deletions (23, 613, and 5 bp) in the first intron between 500 and 1000 bp, one deletion in the fifth intron (70 bp), and six deletions in the sixth intron (40, 5, 13, 29, and 13 bp). In contrast to CAB1, *BoFLC2* in CAB3 and CAB5 each had two deletions (4 and 2 bp) in the sixth intron (between 2500 and 3000 bp) and one deletion (215 bp) in the first intron (between 500 and 100 bp). *BoFLC3* exhibited three analogous deletions ranging from 0 to 1500 bp (2, 2, and 11 bp) in the first intron of each of the three cabbage flowering lines (Figure 1A–C).

A percent identity matrix for *BoFLC1*, *BoFLC2*, and *BoFLC3* was generated by employing CLUSTAL multiple sequence alignment on the *Brassica* species genomic sequences (Tables S1 and S3). Single nucleotide polymorphisms (SNPs) and indels of *FLC* were abundant between cabbage genotypes. In genomic DNA, significant indels in introns were found in all of the *FLC1*, *FLC2*, and *FLC3* genes; though in CDS, it was confirmed that the similarity between each gene was high (Figure 1).

### 3.2. Phylogenetic Relationships among B. oleracea and Other Brassica Species

**Figure 2:**
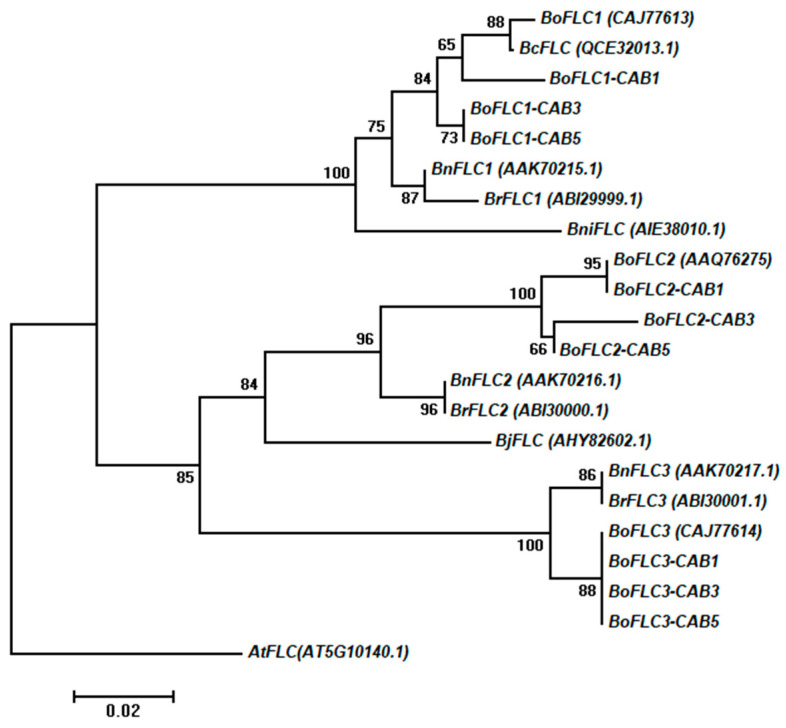
Neighbor-joining tree showing phylogenetic relationships based on decoded *BoFLC* amino acid sequences among early- (CAB1), mid- (CAB3), and late (CAB5)-flowering cabbages. The GenBank accession number is shown in parentheses, and sequences were compared with the three cabbage genotypes used in our study. Bootstrap values ˃ 50% are shown above the branches. *At*, *A. thaliana*; *Bc*, *B. carinata*; *Bj*, *B. juncea*; *Bn*, *B. napus*; *Bni*, *B. nigra*; *Bo*, *B. oleracea*; *Br*, *B. rapa*.

We investigated the relationship between the amino acid sequences (NCBI) of *BoFLC1 (CAJ77613)*, *BoFLC2 (AAQ76275)*, *BoFLC3 (CAJ77614)*, *BrFLC1 (DQ866874.1)*, *BrFLC2 (DQ866875.1)*, *BrFLC3 (ABI30001.1)*, *BnFLC1 (AAK70215.1)*, *BnFLC2 (AAK70216.1)*, *BnFLC3 (AAK70217.1)*, *BniFLC (AIE38010.1)*, *BcFLC (QCE32013.1)*, and *BjFLC (AHY82602.1)* by employing a neighbor-joining tree by protein levels (Figure 2). We used *AtFLC* (*AT5G10140.1*) as an outgroup. *FLC* homologs were distinguishable between *B. oleracea* (*Bo*), *B. rapa* (*Br*), *B. napus* (*Bn*), and *B. nigra*. The *BoFLC1*, *BoFLC2*, and *BoFLC3* proteins of CAB1 were closely related to the known cabbage proteins. CAB3 and CAB5 were highly correlated with *FLC* homology, indicating that most of the correlations between the proteins of the *FLC* homologs of *B. oleracea* were high.

*B. oleracea* plants (Tables S1 and S3), such as cabbage, kohlrabi, and broccoli, showed high genetic similarities, with CAB1-*FLC1* and BR11-*FLC1* exhibiting a homology of 99.1%, and CAB3 and CAB5-*FLC*1 showing a homology of 98.9% with BR11-*FLC1*. The homology between CAB1-*FLC2* and KH8-*FLC2* was very high, reaching 95.2%. Similarly, the homology between CAB3-*FLC*2 and BR11-*FLC2* was 95.2%, while the homology between CAB5-*FLC2* and BR11-*FLC2* was 99.8%. The homology between KH8-*FLC3* and CAB1, CAB3, and CAB5-*FLC3* was 99.1% (Table S3).

### 3.3. Effects of Vernalization on Flowering Repressor (BoFLC1, BoFLC2, and BoFLC3) Expression Profiles

Multiple alignment and phylogenetic investigations determined that CAB3 and CAB5 share structural homology and carry a high number of indels. We thus selected those flowering lines for the gene expression analysis. For *BoFLC*-specific qRT-PCR transcript expression analysis, we created graphs depicting the expression in CAB3 and CAB5 and in the corresponding controls. The controls did not show significant differences in *FLC* homologs. However, the basic expression level in CAB5 was approximately two times higher than that in CAB3 in the control grown in a greenhouse.

**Figure 3:**
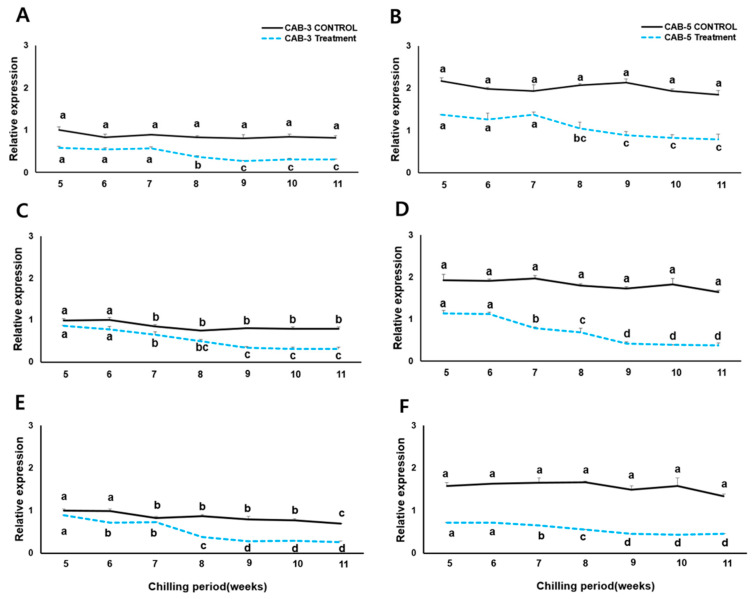
mRNA expression patterns of three *BoFLC* homologs using cDNA from vernalization (dotted lines) and non-vernalization (solid lines) treatments between mid- (CAB 3) and late (CAB 5)- flowering cabbages. Relative expressions of *BoFLC1* (top panels **A**,**B**), *BoFLC2* (middle panels **C**,**D**), and *BoFLC3* (bottom panels **E**,**F**) are shown for CAB 3 (left panels **A**,**C**,**E**, respectively) and CAB 5 (right panels **B**,**D**,**F**, respectively). Different letters indicate statistically significant differences in gene expression across vernalization according to Duncan’s multiple range test at *p* ≤ 0.05.

Investigation of *BoFLC* gene expression in middle- and late-flowering genotypes of cabbage revealed that the *FLC* locus was suppressed in *FLC1*, *FLC2*, and *FLC3* after being vernalized (Figure 3). This treatment significantly reduced the expression related to repression of flowering by approximately 0.5 and 1.0% for *FLC1*, by 0.5 and 1.5% for *FLC2*, and by 0.5 and 1.2% for *FLC3* in CAB3 and CAB5, respectively. Our experimental results confirmed that *BoFLC1* expression decreased rapidly in CAB3 and CAB5 following vernalization for approximately 8 weeks (Figure 3A,B). However, the expression did not continue to decrease, and *BoFLC1* expression was maintained for 9 weeks in both cabbages under the vernalization treatment. *BoFLC2* expression in both CAB3 and CAB5 cabbages began to decrease after approximately 7 weeks of vernalization (Figure 3C,D), and the expression values were maintained until 9 weeks. *BoFLC3* in CAB5 showed identical suppression at 8 weeks, although vernalized plants exhibited substantial declines in expression starting from the 6th week (Figure 3E,F).

### 3.4. Effects of Vernalization on the Expression Profile of Flowering Regulators (BoGI, BoCOOLAIR, and BoVIN3)

**Figure 4:**
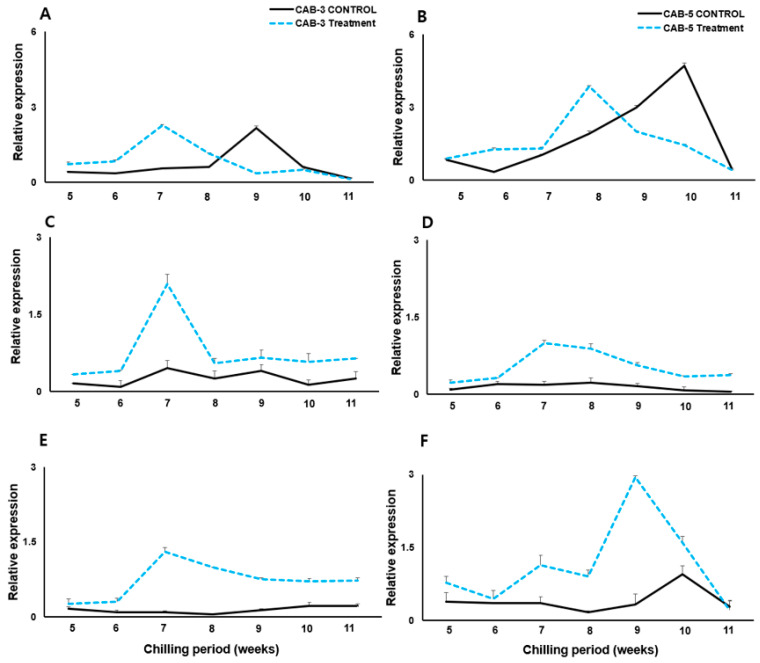
mRNA expression patterns of the flowering regulator genes *BoGI* (*GI*, top panel **A**,**B**), *BoCOOLAIR* (middle panel **C**,**D**), and *BoVIN3* (bottom panel **E**,**F**) between mid- (CAB 3) and late (CAB 5)-flowering cabbage lines under vernalization (dotted lines) and non-vernalization (solid lines) treatments. The relative expressions of *GI*, *BoCOOLAIR*, and *BoVIN3* were compared for the mid-flowering CAB 3 line (left **A**,**C**,**E**) and late-flowering CAB 5 line (right **B**,**D**,**F**). The bars indicate standard errors of the means for each week of the vernalization treatment.

We also performed qRT-PCR tests on *BoGI*, *BoCOOLAIR*, and *BoVIN3* to explore the expression of genes related to flowering in CAB3 (Figure 4A,C,E), CAB5 (Figure 4B,D,F), and controls. The expression of *BoGI* in CAB3 peaked (2.8%; Figure 4A) at 7 weeks, whereas the control showed the highest expression at 9 weeks. The peak appeared approximately 2 weeks faster than for the control. For CAB5, the control exhibited the highest peak (5.0%) at 10 weeks (Figure 4B), while the vernalization-treated cabbage showed the highest peak (4.0%) at 8 weeks.

In terms of *COOLAIR* expression, both CAB3 (Figure 4C) and CAB5 (Figure 4D) under the vernalization treatment showed the highest expression levels at 7 weeks. This coincided with the time when the expression of the *BoFLC* gene began to decrease. In CAB3, *COOLAIR* increased rapidly at week 7, up to two-fold, and decreased rapidly thereafter. In contrast, in CAB5 (Figure 4D), *COOLAIR* increased more slowly, up to approximately 1.3-fold, and then gradually decreased. The CAB3 control showed the highest relative expression (approximately 0.4%) at 10 weeks, after which it decreased. In contrast, the CAB5 control showed a maximum peak at 8 weeks (0.1%), after which the expression slowly decreased.

The highest expression of *BoVIN3* in vernalization-treated cabbages was observed at 7 weeks in CAB3 (Figure 4E) and at 9 weeks in CAB5 (Figure 4F), with expressions that were approximately 1.5- and 3-fold increased, respectively. In CAB3, expression slowly decreased after 7 weeks but was maintained after 9 weeks, whereas in CAB5 expression decreased continuously after 9 weeks. The CAB3 control showed the highest relative expression (0.2%) at 10 weeks, which was maintained until week 11. The CAB5 control showed a maximum peak at 10 weeks (1.0%), but expression immediately decreased thereafter.

This study employed a systematic approach to investigate the molecular mechanisms underlying the differential flowering behavior of cabbage genotypes (CAB3 and CAB5). This study examined the impact of vernalization-induced suppression of *FLC* loci in exclusively *BoFLC1*, *BoFLC2*, and *BoFLC3*, as well as the concurrent upregulation of flowering regulators such as *BoGI*, *BoCOOLAIR*, and *BoVIN3*.

## 4. Discussion

Vernalization naturally stimulates flowering in several plant species by exposing them to cold temperatures. Vernalization expedites the process of flowering by facilitating the transition from the growth of leaves and stems to the production of flowers. The organs that emerge from the shoot apex during this transition are determined by genes that regulate the characteristics of the shoot apical meristem. Throughout vegetative growth, the shoot apical meristem generates leaf primordia. During the initiation of reproductive development, the shoot apex transforms into an inflorescence meristem. Additionally, further meristems called flower primordia begin to emerge above each leaf primordium for the formation of different flower parts [36,37,38,39].

Floral repressors, such as *FLC*, serve to inhibit flowering, thus ensuring accurate reproductive timing and mitigating adverse effects on the reproductive success of plants. They establish complexes that coordinate responses to endogenous and environmental stimuli through interaction with inductive pathways. The decrease in *FLC* activity plays a crucial role in the vernalization response in plants. *FLC* plays a role in regulating the flowering response of plants exposed to vernalizing conditions (extended cold) [40,41,42]. Long-term cold initiates vernalization by causing *FLC* to be epigenetically silenced.

Li et al. [43] investigated the impact of vernalization on the flowering responses of *B. oleracea* var. *capitata* and *A. thaliana* by studying the *FLC* locus. The authors conducted a comprehensive investigation on the impact of a 215 bp insertion or deletion (indel) at intron I of the *BoFLC2* gene in *B. oleracea* var. *capitata* during the process of vernalization-induced flowering. Okazaki et al. [11] examined and described the *BoFLC* gene and identified quantitative trait loci associated with flowering. Uptmoor et al. [44] developed a phenology model for predicting the flowering period of *B. oleracea* based on quantitative trait loci (QTL). Multiple researchers have investigated the impact of vernalization on the flowering responses mediated by *FLC* in various plant species, including *A. thaliana* [45,46,47,48,49,50,51,52], *A. lyrate* [53], *B. rapa* [16] *B. napus* [35], *Sinapis alba* [54], *Beta vulgaris* [55], and *Triticum aestivum* [56]. Among the many genes that impact flowering, *FLC* is the primary gene. The repressive effect of *FLC* on flowering in plants is consistent; however, vernalization introduces many variations in the action of *FLC*. *FLC* has frequent allelic variation, and its homologs have been considered strong candidates for regulating flowering time in other Brassicaceae species [57,58,59,60,61,62,63,64,65].

**Figure 5:**
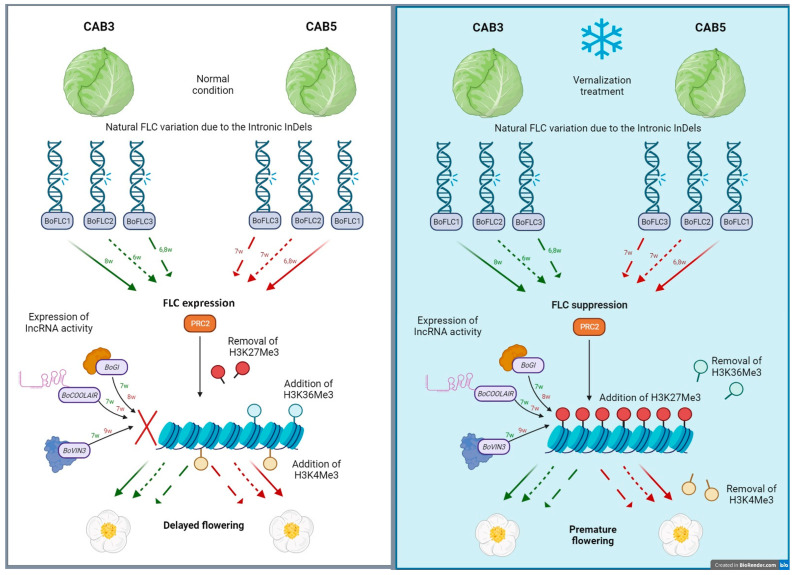
Schematic representation of *FLC* expression and suppression under control and vernalization conditions along with *FLC* regulator activity on three different *BoFLC* genes in CAB3 and CAB5 cabbages (w = weeks; green color arrows = CAB3 *FLC* expressions; red color arrows = CAB5 *FLC* expressions; maximum dotted lines = expression of *BoFLC2*; minimal dotted lines = expression of *BoFLC3*; solid lines = expression of *BoFLC1*).

Our gene structure prediction uncovered the facts of the above studies in three *FLC* homologs—*BoFLC1*, *BoFLC2*, and *BoFLC3*—in three distinct flowering cultivars of *B. oleracea* var. *capitata*, with the indel aligned against the reference genome *AM231517.1* (Figure 5). We found that, out of the three distinct flowering lines, the medium- (CAB3) and late (CAB5)- flowering cabbage lines had the highest rates of insertion and deletion in the *BoFL1* gene, affecting the first, second, fifth, and sixth introns of those genes, respectively. For *BoFLC2*, both CAB3 and CAB5 had high indel levels in the first and sixth introns; CAB3 had two additional insertions. Although all flowering lines had the same indel in the *BoFLC3* gene at the first intron, an extra insertion was found in the fifth intron of CAB3. These findings line up with the Li et al. [43] who mentioned as intron-specific mutations (insertion and deletion) in *FLC* may significantly modify cabbage flowering. We thus chose CAB3 and CAB5 as the maximum indel lines for measuring gene expression while they were subjected to vernalization. Based on indel identification, we speculate that the genotypical changes between CAB3 and CAB5, which had severe mutations in the respective *FLC* homologs, might be an important reason for the delayed flowering of these respective cabbage varieties. We believe that the unique mutation patterns in *BoFLC2* and *BoFLC3* may dramatically affect cabbage flowering. 

Antisense *COOLAIR* is crucial for the coordinated switching of chromatin states in *FLC* during vernalization, a mechanism that connects transcriptional repression with epigenetic silencing through decreasing H3K36/K4 methylation and increasing H3K27me3 at the intragenic *FLC* nucleation site. Two proteins, VERNALIZATION 2 (VRN2) and VIN3, are also involved in the epigenetic suppression of *FLC* expression during vernalization. This mechanism of action is critical for comprehending the involvement of *COOLAIR* in the vernalization and epigenetic silencing of *FLC* [59,66].

*COOLAIR* IncRNAs exert functional repression of *FLC* sense expression during the early stages of cold exposure (vernalization) via many mechanisms, including direct association with *FLC* chromatin and modulation of H3K36me3, H3K4me3, and H3K27me3 levels. *COOLAIR* begins immediately after the primary sense transcript poly(A) site and ends either prematurely or continues into the *FLC* promoter region. Splicing of *COOLAIR* plays a crucial role in its functionality [67,68,69,70,71]. Several studies have examined the varying flowering responses resulting from intronic mutations in plants: the AG-to-AA mutation (*COOLAIR^AA^*) markedly reduces the splicing efficiency of the intron and increases the levels of *FLC* expression [67,68,69,70,71]. The naturally occurring large insertion in the first intron of *BrFLC2* and *BrFLC*3 has been extensively studied [72]. This insertion leads to a weak suppression of cold sensitivity, which was demonstrated on the exceptionally delayed bolting cultivar (Tsukena No. 2) of *B. rapa* L. ssp. *pekinensis* (Chinese cabbage). Novel MADS-box genes and very late blooming variants were produced by intronic mutations such as insertion and deletion in *A. arenosa* [73,74].

Our findings showed several insertions and deletions in the *BoFLC* locus in different flowering cabbage genotypes, particularly in the first, fifth, and sixth introns. We assume that this might be an important factor in the varied flowering patterns through its influence on *BoFLC* and antisense *BoCOOLAIR* expression levels among the cabbages as Li et al. [43] identified.

A phylogenetic investigation by Okazaki et al. [11] demonstrated that the *BoFLC2* clone in *B. oleracea*, which is a member of the *FLC*2 clade within the *Brassica* family, is 91% identical to *BrFLC2*. In addition, Lin et al. [75] demonstrated that *BoFLC*4 had a high degree of similarity with *BrFLC4*, at 98%. Schranz et al. [20] found three *Brassica FLC* homologs in *B. oleracea*: *BoFLC1*, *BoFLC3*, and *BoFLC5*. Our study confirms the high homology of *BoFLC1*, *BoFLC2*, and *BoFLC3* in CAB1, CAB3, and CAB5 with the respective *FLC* homologs of *B. rapa*, *B. napus*, and *B. nigra* varieties. The percent identity matrix of the CDS multiple sequence alignment revealed a significant degree of sequence similarity across the various flowering *B. oleracea* plants (early, middle, and late flowering).

Our analysis of gene expression in both vernalized and non-vernalized forms of CAB3 and CAB5 revealed a significant decrease in the expression of all *BoFLC* genes, including *BoFLC1*, 2, and 3, under vernalized conditions. Furthermore, the drop in expression was particularly pronounced in all *BoFLC* loci in the CAB5 cabbage. In both CAB3 and CAB5, *BoFLC1* expression decreased during the seventh week of vernalization, whereas that of *BoFLC2* and *BoFLC3* decreased during the sixth week. This time-dependent reduction in gene expression over time may be explained by sequence similarity of the corresponding gene (*BoFLC1*~3) CDS between the two cabbage lines, as well as the mutations in introns, which were validated using phylogenetic and multiple sequence alignment studies. Numerous investigations [76,77,78] have shown that the course of vernalization in *Brassica* species has a notable impact on flowering. Our investigation also indicates that the suppression of *BoFLC* genes plays a crucial role in this phenomenon. There is a suspicion that the presence of two insertions in the sixth intron of *BoFLC2* and one insertion in the fifth intron of *BoFLC3* of CAB3 may have contributed to the minimized suppression of *BoFLC* in CAB3. This suspicion is based on the observation that the suppression of *FLC* was more pronounced in CAB5 compared to CAB3. Several studies [33,59,61,67,68,69,70,71,79,80] in *Arabidopsis* and other Brassicaceae addressed similar results.

The alteration of chromatin structure by lncRNAs could be influenced both inside and beyond their transcriptional unit by base pairing, triplex formation with DNA or RNA, or under the guidance of proteins. The co-transcriptional regulation (cis) of *COOLAIR* in a large number of cells strongly induces the suppression of sense *FLC* transcription through a distinct interaction. The overexpression of *COOLAIR* occurs simultaneously with the suppression of a gene loop at *FLC*. This leads to maximum intragenic polycomb nucleation and an accelerated shutdown of sense transcription within 1 to 4 weeks. Antisense transcription and the existence of clusters of cis-tethered transcripts efficiently suppress the transcription of *FLC*. The repression of *FLC* transcription is essential for enabling polycomb-induced epigenetic switching [61,67,68,69,70,71].

The *FLC* locus produces two main types of *COOLAIR* isoforms through alternative splicing of antisense transcripts. These isoforms terminate at either the proximal site (sense intron 6, class I) or the distal site (sense promoter, class II) of the *FLC* locus. The expression of class I *COOLAIR* RNAs exhibits a more pronounced rate of rise compared to class II *COOLAIR* RNAs during vernalization [33,59,79,80]

Previous studies [76] have extensively documented the significance of alterations in *FLC* and *COOLAIR* expression, which result in variations in flowering. AtNDX, a homeodomain protein that regulates *COOLAIR*, is a set of antisense transcripts derived from the 3′ end of the *Arabidopsis FLC*. AtNDX binds to single-stranded DNA but not double-stranded DNA non-sequences in vitro and localizes to the heterochromatic region of the *COOLAIR* promoter in vivo. Single-stranded DNA has been detected in vivo as part of an RNA–DNA hybrid or R-loop covering the *COOLAIR* promoter. AtNDX-mediated R-loop stabilization represses *COOLAIR* transcription, which in turn modifies *FLC* expression. However, if the R-loop is not stable, *COOLAIR* cannot be inhibited, ultimately leading to a decrease in *BoFLC* expression. If differences in AtNDX-mediated R-loop stabilization occur due to differences in the introns of *BoFLC*, this will be an important factor in *FLC* expression.

According to Hung et al. [81], *COOLAIR* is an extended noncoding RNA (lncRNA) that is transcribed from the 3′ stop of the *FLC* gene in the opposite direction. *COOLAIR* can repress *FLC* expression by increasing histone H3 lysine 27 trimethylation (H3K27me3) levels at the *FLC* chromatin. However, *COOLAIR* is not always an exact antisense reproduction of the *FLC* gene, as it has different transcription sites (TSSs) and opportunity splicing patterns. Therefore, *COOLAIR* will have altered binding to the *FLC* locus depending on the series versions between one-of-a-kind flowering biotypes. For example, the *FRI* allele can have an effect on the TSSs and splicing of *COOLAIR*, resulting in special *COOLAIR* isoforms which have exceptional consequences on *FLC* repression. The authors additionally cited that WRKY63, a transcription factor, can bind to one-of-a-kind regions of the *FLC* locus and set off *COOLAIR* expression undern vernalization. These elements might also contribute to the altered binding of *COOLAIR* to the *FLC* locus. This transcriptional activation of flowering regulators such as *COOLAIR* and *COLDAIR* regulates vernalization-induced flowering [81].

Vernalized CAB3 and CAB5 exhibited early peak expression of *BoGI* for two weeks, whereas CAB3 and CAB5 displayed early peak expression of *BoVIN3* for four and one week, respectively, about *BoGI*, *BoCOOLAIR*, and *BoVIN3* compared to the control. *BoCOOLAIR* expression increased most significantly in CAB3, while *BoVIN3* expression increased the most in CAB3 and CAB5 compared to the control. We obtained intriguing results by comparing expression levels of regulators (antisense of repressors) and flowering repressors. The precise time required for *BoFLC* to decrease was determined by the process of vernalization, which correlatedly upregulate the antisense regulators *BoCOOLAIR*, along with *BoGI*, and *BoVIN3*. This supports the importance of the cold condition, which significantly enhanced the expression of *BoGI* and *BoVIN3* in the corresponding flowering cabbages, and highlights the strong correlation between flowering repressors and regulator expression during vernalization. This result supports the previous findings of the antisense-mediated suppression of *FLC* [33,58,79].

Our investigation determined that the CAB1 cabbage variety had the lowest number of genetic changes in comparison to the other two cabbage genotypes CAB3 and CAB5. Despite the difference in flowering times between CAB3 and CAB5 cabbage genotypes, their gene structures are similar, with the exception of two more insertions in the sixth intron of the *BoFLC2* gene and one additional insertion in the fourth intron of the *BoFLC3* gene in CAB3. Subsequently, we conducted vernalization-mediated gene expression studies on these two cabbage genotypes to examine the distinct flowering patterns seen in cabbages with comparable genetic makeups. Research results showed that CAB5 exhibited higher downregulation of all three *BoFLC* genes compared to CAB3. Even though the gene expression investigations of three flowering regulators (*BoGI*, *BoCOOLAIR*, and *BoVIN3*) revealed that CAB5 had the most significant influence on the expression of *BoGI* and *BoVIN3*, the comparatively earlier-flowering cabbage genotype CAB3 had the greatest impact (approximately two-fold) on *BoCOOLAIR* expression, which is ultimately needed for the suppression of *BoFLC*. Therefore, with all the facts mentioned above, we hypothesize that *BoCOOLAIR* likely had a significant influence on impeding *BoFLC* in the genetic variation of late-flowering and medium-flowering cabbage genotypes. In addition, we anticipate that the integration of three additional insertions into CAB3 might have contributed to the elevation of *BoCOOLAIR* levels and the inhibition of *FLC*. This work serves as a basis for future studies that aim to design cabbage varieties by modifying flowering in diverse Brassicaceae types using *BoFLC*-specific genome-editing techniques such as CRISPR-CAS9. Furthermore, markers for the selection of cultivars with varying blooming durations might be produced.

## Data Availability

Data are contained within the article or Supplementary Material.

## Supplementary Materials

The following supporting information can be downloaded at https://www.mdpi.com/article/10.3390/genes15020154/s1 and the National Agricultural Biotechnology Information Center (NABIC; https://nabic.rda.go.kr/ (accessed on 20 April 2020)). Table S1: List of cabbage, kohlrabi, and broccoli materials used in this study with their flowering time; Table S2: List of primers used in the molecular assessment of this study. Table S3: Percent identity matrix of CDS multiple sequence alignment for cabbages (*FLC1*-CA1, -CA3, and -CA5), kohlrabi (*FLC2*-KH7 and -KH8), and broccoli (*FLC3*-BR10 and -BR11) with different flowering times.
