# Supplementary material for: Molecular Evaluation of the Effects of FLC Homologs and Coordinating Regulators on the Flowering Responses to Vernalization in Cabbage (Brassica oleracea var. capitata) Genotypes
Source: Genes (Basel). 2024 Jan 24;15(2):154. doi: 10.3390/genes15020154 (PMC10887945; doi:10.3390/genes15020154)
Supplement: Supplementary file 1 [file genes-15-00154-s001.zip › genes-2747378-supplementary.pdf]

Table S1. List of cabbage, kohlrabi, and broccoli materials used in this study with their flowering time.

Species	Common name	Genotype name	Flowering type
B. oleracea var. *capitata* (BOC)	Cabbage	20*FLC*-CAB1	Early
		20*FLC*-CAB3	Medium
		20*FLC*-CAB5	Late
B. oleracea var. *gongylodes* (BOG)	Kohlrabi	20*FLC*-KH7	Late
		20*FLC*-KH8	Early
B. oleracea var. *italica* (BOI)	Broccoli	20*FLC*-BR10	Early
		20*FLC*-BR11	Late

Table S2. List of primers used in this molecular assessment of this study.

Purpose	Gene identification no.	Gene name	Forward primer	Sequence (5'-3')	Reverse primer	Sequence (5'-3')
Genomic DNA cloning	*Bo9g173400*	*BoFLC1*	*BoFLC1*-gF	AGATCAAATTAGGGCGCAAAGC	*BoFLC1*-gR	ATTCGCCGATTAAAGGTAAC
	Bo3g100540	*BoFLC2*	*BoFLC2*-gF	ACCGAACCGAACCTCAGGATC	*BoFLC2*-gR	GTAGTTTTTACACACGGGGTC
	Bo3g005470	*BoFLC3*	*BoFLC3*-gF	CACTTGAACCGAACCTCTGG	*BoFLC3*-gR	ATTTCAGCCCCGTCTAAAGG
Genomic DNA cloning (Contig)	*Bo9g173400*	*BoFLC1*	*BoFLC1*-F1	CGCTGTGAGGTTGATGCT	*BoFLC1*-R1	AGCATCAACCTCACAGCG
			BoFLC1-F2	CCAAGCGTTTAGTTTCGC	*BoFLC1*-R2	GCGAAACTAAACGCTTGG
			BoFLC1-F3	ACGATTGCGTTTGCATGT	*BoFLC1*-R3	ACATGCAAACGCAATCGT
			BoFLC1-F4	ATGTATTGGCATGCCCGC	*BoFLC1*-R4	GCGGGCATGCCAATACAT
			BoFLC1-F5	AGTCTGCTTCATGAGGCC	*BoFLC1*-R5	GGCCTCATGAAGCAGACT
	Bo3g100540	*BoFLC2*	*BoFLC2*-F1	GAGCAATGTCCTGATGAGC	*BoFLC2*-R1	GCTCATCAGGACATTGCTC
			BoFLC2-F2	ATTGTGCAGCCATTAACC	*BoFLC2*-R2	GGTTAATGGCTGCACAAT
			BoFLC2-F3	TGGTTCACACCATGAGCTAC	*BoFLC2*-R3	GTAGCTCATGGTGTGAACCA
			BoFLC2-F4	GAACCAGGGTTTGGCTAG	*BoFLC2*-R4	CTAGCCAAACCCTGGTTC
			BoFLC2-F5	AATGAAGCCTGGATCTGG	*BoFLC2*-R5	CCAGATCCAGGCTTCATT
	Bo3g005470	*BoFLC3*	*BoFLC3*-F1	TTTGCATGTCCGTCAAGA	*BoFLC3*-R1	TCTTGACGGACATGCAAA
			BoFLC3-F2	AGGAACAAGCATTCCTTTTC	*BoFLC3*-R2	GAAAAGGAATGCTTGTTCCT
			BoFLC3-F3	GCCAACTCATAGCCTCAA	*BoFLC3*-R3	TTGAGGCTATGAGTTGGC
			BoFLC3-F4	TGGAGGGTGTCCTTGAGA	*BoFLC3*-R4	TCTCAAGGACACCCTCCA
			BoFLC3-F5	GAAGAAGAAACAACCAAGGC	*BoFLC3*-R5	GCCTTGGTTGTTTCTTCTTC
Expression analysis	*Bo9g173400*	*BoFLC1*	*BoFLC1*-F	CGATAATGTAAGTGTGGGTTCC	*BoFLC1*-R	GGAGATTTGTCCAGGTGACACC
	Bo3g100540	*BoFLC2*	*BoFLC2*-F	TCTGATGTAAGCGTCGATTCC	*BoFLC2*-R	CGGACAATTGATGTCAGAGATC
	Bo3g005470	*BoFLC3*	*BoFLC3*-F	GAGCTATGGTTCACACAATGAGTTAC	*BoFLC3*-R	GGCTATCAACAAGCTTCAACATTAG
	Bo5g038860	*BoGI*	*BoGI*-F	TGGCAAGTGCATCTGATCTC	*BoGI*-R	TGGGTGTGATAAGCACCGTA
	Bo9g173400	*BoCOOLAIR*	*BoCOOLAIR*-F	TGATGGTTGGTAGGAAAAGA	*BoCOOLAIR*-R	CCGCAAGATTATTCTTCTCCA
	Bo3g019340	*BoVIN3*	*BoVIN3*-F	TGGATTGCTTGTGAGGGTTGT	*BoVIN3*-R	TACATGCCGAGTCTCCTTCG
	Bol030974	*BoACTIN1*	*BoACTIN1*-F	TTCTCTCTTCCACACGCCAT	*BoACTIN1*-R	CTTGTCCTGCGGGTAATTCG

Table S3. Percent identity matrix of CDS multiple sequence alignment cabbages (*FLC1*-CAB1, -CAB3, and -CAB5), kohlrabi (*FLC2*-KH7 and -KH8), and broccoli (*FLC3*-BR10 and –BR11) having different flowering times.

	***FLC1*-CAB1**	***FLC1*-CAB3**	***FLC1*-CAB5**	***FLC1*-KH7**	***FLC1*-KH8**	***FLC1*-BR10**	***FLC1*-BR11**	***FLC2*-CAB1**	***FLC2*-CAB3**	***FLC2*-CAB5**	***FLC2*-KH7**	***FLC2*-KH8**	***FLC2*-BR10**	***FLC2*-BR11**	***FLC3*-CAB1**	***FLC3*-CAB3**	***FLC3*-CAB5**	***FLC3*-KH7**	***FLC3*-KH8**	***FLC3*-BR10**	***FLC3*-BR11**
FLC1-CAB1	100	98.48	98.48	98.65	98.69	93.27	99.16														
FLC1-CAB3		100	100	99.82	98.69	93.27	98.99														
FLC1-CAB5			100	99.82	98.69	93.27	98.99														
FLC1-KH7				100	99.82	94.13	98.84														
FLC1-KH8					100	94.29	98.72														
FLC1-BR10						100	93.58														
FLC1-BR11							100														
FLC2-CAB1								100	94.92	99.49	98.99	99.49	98.98	99.32							
FLC2-CAB3									100	95.42	94.92	94.42	94.91	95.25							
FLC2-CAB5										100	99.66	98.98	99.49	99.83							
FLC2-KH7											100	98.48	99.15	99.49							
FLC2-KH8												100	98.48	98.82							
FLC2-BR10													100	99.66							
FLC2-BR11														100							
FLC3-CAB1															100	100	100	100	99.15	93.94	96.62
FLC3-CAB3																100	100	100	99.15	93.94	96.62
FLC3-CAB5																	100	100	99.15	93.94	96.62
FLC3-KH7																		100	99.15	93.89	96.59
FLC3-KH8																			100	93.72	96.42
FLC3-BR10																				100	96.29
FLC3-BR11																					100
